# The urban features of informal settlements in Jakarta, Indonesia

**DOI:** 10.1016/j.dib.2017.10.049

**Published:** 2017-10-24

**Authors:** Waleed Alzamil

**Affiliations:** College of Architecture and Planning, King Saud University, Saudi Arabia

**Keywords:** Informal settlements, Physical, Features, Urban, Kampung, Jakarta, Indonesia

## Abstract

This data article contains the urban features of three informal settlements in Jakarta: A. Kampung Bandan; B. Kampung Luar Batang; And C. Kampung Muara Baru. The data describes the urban features of physical structures, infrastructures, and public services. These data include maps showing locations of these settlements, photography of urban status, and examples of urban fabric. The data are obtained from the statistical records and field surveys of three settlements cases.

**Specifications table**

**Table:** 

Subject area	*Urban planning, architecture, urbanism*
More specific subject area	*Housing, urban renewal*
Type of data	*Maps, tables, charts, photographs*
How data was acquired	Field survey
	Field investigation with residents
	Review of reports
Data format	*Raw and analyzed*
Experimental factors	*Descriptive analysis, Comparative analysis*
Experimental features	*Analysis the most important features of these settlements based on respondents’ views.*
Data source location	*Jakarta, Indonesia*
Data accessibility	*Data is presented with this article*

**Value of the data**

• The data shows the urban status of informal settlements in Jakarta.
• Data show the actual needs of low-income households in the urban environment.
• The data helps to know the preferences of the residents on the urban environment.

## 1 Data

**Fig. 1:**
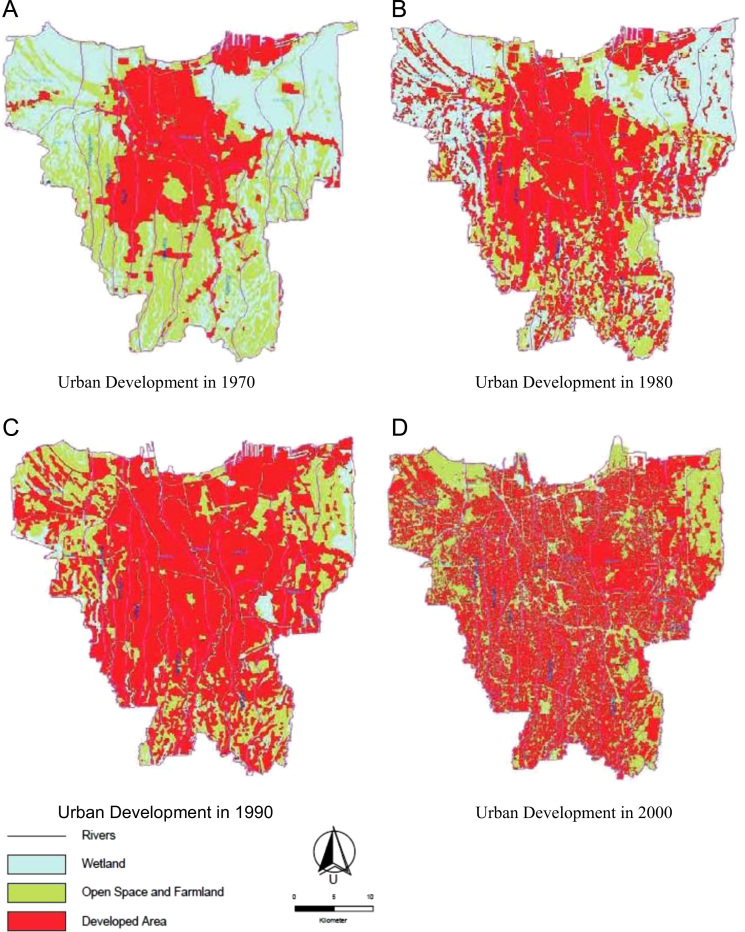
The stages of urban development in Jakarta [1].

**Fig. 2:**
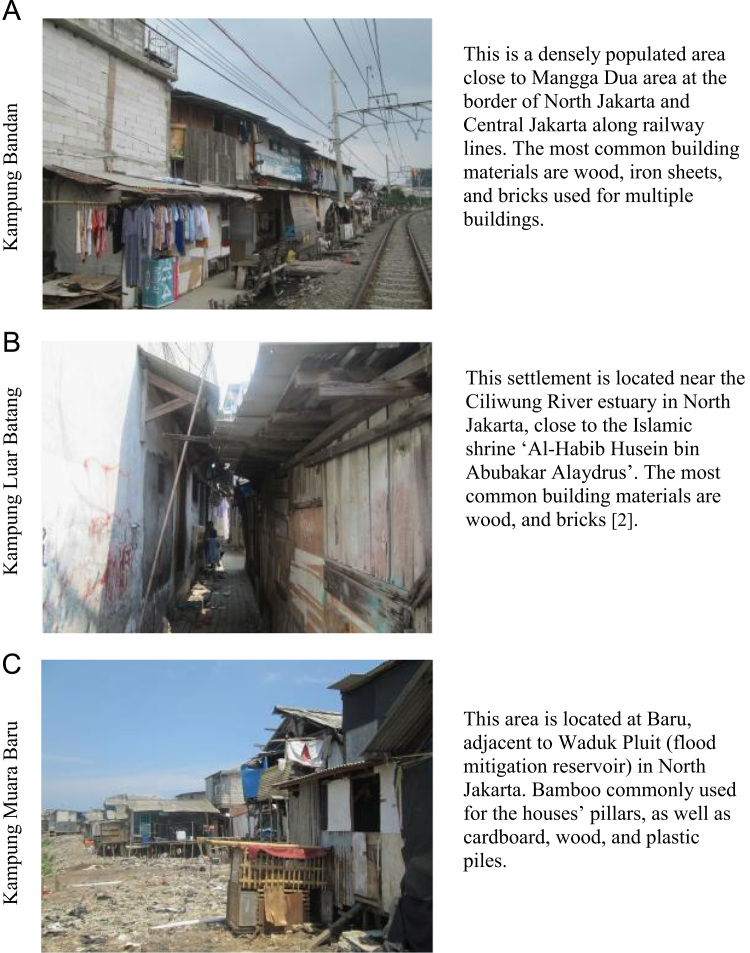
The urban features of informal settlements in Jakarta. Source: Survey by the author.

The data presented include population growth in Jakarta (Table 1), the urban expansion in Jakarta since 1970 (Fig. 1), and the urban features of three informal areas in Jakarta (Fig. 2).

**Table 1:** Population growth in Jakarta [2].

Year	Population	Growth	Growth rate
1950	1,452,000	0	0.00%
1955	1,972,000	520,000	35.80%
1960	2,679,000	707,000	35.90%
1965	3,297,000	618,000	23.10%
1970	3,915,000	618,000	18.70%
1975	4,813,000	898,000	22.90%
1980	5,984,000	1,171,000	24.30%
1985	7,009,000	1,025,000	17.10%
1990	8,175,000	1,166,000	16.60%
1995	8,322,000	147,000	1.80%
2000	8,390,000	68,000	0.80%
2005	8,988,000	598,000	7.10%
2010	9,630,000	642,000	7.10%
2015	10,323,000	693,000	7.20%
2017	10,660,000	337,000	3.30%
2020	11,298,000	638,000	6.00%
2025	12,589,000	1,291,000	11.40%
2030	13,812,000	1,223,000	9.70%

## 2 Experimental design, materials and methods

Rapid urbanization and population growth is the main reason for the emergence of informal settlements in Jakarta. The massive urbanization contributed to the shortage of land supply and high land values. As a result, more than 25% of agricultural uses have been converted into industrial, commercial, or residential uses to meet the growing demand for land [1]. Analysis of the respondents’ views on physical features examines housing conditions, housing spaces, structural problems, housing services, construction density, lighting and ventilation. Also, the analysis describes the respondents’ views on infrastructures such as electricity connections, water supply, rainwater drainage, waste water sewage, and waste collection. Finally, the analysis describes the respondents’ views on public services such as the quality and availability of shops, schools, parks, children's playgrounds, public spaces, health services, and security services (Table 2, Table 3).

**Table 2:** Respondents’ views on the physical features of informal settlements in Jakarta. Source: Survey by the author.

Physical status	Respondents																								
	1	2	3	4	5	6	7	8	9	10	11	12	13	14	15	16	17	18	19	20	21	22	23	24	25
**A. Kampung Bandan**																									
Deteriorating housing	✓	✓	✓	✓	✓	✓	✓	✓	✓	✗	✓	✗	✓	✓	✓	✗	✓	✗	✓	✓	✓	✓	✗	✗	✓
Shortage of housing spaces	✓	✓	✓	✓	✓	✓	✓	✗	✗	✗	✓	✓	✗	✓	✗	✓	✗	✓	✓	✓	✓	✓	✗	✓	✗
Lack of housing services	✓	✓	✓	✗	✗	✓	✓	✓	✓	✗	✗	✓	✗	✗	✗	✓	✓	✓	✓	✓	✓	✓	✓	✓	✗
Temporary construction materials	✗	✗	✗	✗	✗	✗	✗	✗	✓	✗	✗	✗	✗	✓	✗	✓	✗	✗	✗	✗	✗	✗	✗	✗	✗
Lighting and ventilation	✗	✗	✗	✓	✓	✗	✗	✓	✗	✗	✗	✗	✓	✗	✗	✗	✗	✓	✗	✗	✗	✗	✗	✗	✗
Structural problems	✗	✗	✗	✗	✗	✗	✗	✗	✗	✗	✓	✗	✗	✗	✗	✓	✗	✗	✗	✗	✗	✗	✗	✗	✗
Abandoned buildings	✗	✗	✗	✗	✗	✗	✗	✗	✗	✓	✗	✗	✗	✗	✗	✗	✓	✗	✗	✗	✗	✗	✗	✗	✗
High-density construction	✗	✗	✗	✗	✗	✗	✗	✗	✗	✗	✗	✗	✗	✗	✗	✗	✗	✗	✗	✗	✗	✗	✗	✓	✓
Others	✗	✗	✗	✗	✗	✗	✗	✗	✗	✗	✗	✓	✓	✗	✗	✗	✗	✗	✗	✗	✗	✗	✗	✗	✗
**B. Kampung Luar Batang**																									
Deteriorating housing	✓	✓	✗	✗	✓	✗	✓	✓	✓	✓	✗	✓	✗	✗	✓										
Shortage of housing spaces	✓	✓	✓	✓	✗	✓	✗	✗	✓	✗	✗	✗	✓	✓	✓										
Lack of housing services	✓	✓	✗	✓	✓	✗	✓	✗	✗	✗	✓	✗	✓	✓	✗										
Temporary construction materials	✗	✗	✓	✗	✓	✗	✓	✗	✗	✗	✗	✓	✗	✗	✗										
Lighting and ventilation	✗	✗	✗	✗	✓	✓	✗	✗	✗	✗	✗	✗	✗	✗	✗										
Structural problems	✗	✗	✓	✗	✓	✓	✗	✗	✗	✗	✗	✗	✗	✗	✗										
Abandoned buildings	✗	✗	✗	✗	✗	✗	✗	✗	✗	✗	✓	✗	✓	✗	✗										
High-density construction	✗	✗	✗	✗	✗	✗	✗	✗	✗	✗	✗	✗	✗	✗	✗										
Others	✗	✗	✗	✗	✗	✗	✗	✗	✗	✗	✗	✗	✗	✗	✗										
**C. Kampung Muara Baru**																									
Deteriorating housing	✗	✗	✗	✓	✓	✓	✗	✓	✗	✗	✗	✓	✗	✓	✗	✓	✓	✓	✓	✓					
Shortage of housing spaces	✗	✓	✗	✓	✗	✓	✗	✓	✗	✗	✓	✓	✓	✗	✓	✓	✓	✓	✓	✓					
Lack of housing services	✗	✗	✓	✓	✗	✗	✓	✓	✓	✓	✗	✗	✓	✗	✓	✗	✗	✗	✓	✓					
Temporary construction materials	✗	✗	✗	✓	✗	✗	✓	✗	✗	✓	✗	✓	✓	✗	✓	✓	✗	✓	✗	✗					
Lighting and ventilation	✓	✓	✓	✗	✗	✗	✗	✗	✓	✓	✗	✗	✗	✗	✗	✗	✗	✗	✗	✗					
Structural problems	✗	✓	✗	✗	✗	✗	✗	✗	✗	✗	✓	✗	✗	✓	✗	✗	✓	✓	✗	✗					
Abandoned buildings	✗	✗	✗	✗	✗	✗	✗	✗	✓	✗	✗	✗	✗	✓	✗	✗	✗	✗	✗	✗					
High-density construction	✗	✗	✓	✗	✗	✗	✗	✗	✗	✗	✓	✗	✗	✗	✗	✗	✗	✗	✗	✗					
Others	✗	✗	✗	✓	✗	✗	✗	✗	✗	✗	✗	✗	✗	✗	✗	✗	✗	✗	✗	✗					

✓: Responder agrees with this feature.

✗: Responder disagrees with this feature.

**Table 3:** Respondents’ views on the utility features of informal settlements in Jakarta. Source: Survey by the author.

Utility status	Respondents																								
	1	2	3	4	5	6	7	8	9	10	11	12	13	14	15	16	17	18	19	20	21	22	23	24	25
**D. Kampung Bandan**																									
Forms of water supply	✓	✓	✓	✓	✓	✓	✓	✗	✗	✓	✓	✓	✓	✓	✗	✓	✗	✓	✗	✗	✗	✗	✗	✗	✓
Waste water sewage	✓	✗	✗	✗	✗	✗	✗	✓	✓	✗	✓	✓	✗	✗	✗	✓	✗	✓	✗	✗	✗	✗	✗	✗	✗
Problems with waste collection	✗	✗	✗	✓	✗	✓	✗	✓	✓	✗	✓	✗	✗	✗	✓	✓	✗	✗	✗	✗	✗	✗	✗	✓	✗
Forms of electricity connection	✓	✗	✗	✗	✓	✗	✗	✗	✗	✗	✗	✗	✓	✓	✗	✓	✗	✓	✗	✗	✗	✗	✗	✓	✗
Cable, internet, telephone, TV	✗	✗	✓	✗	✗	✓	✓	✓	✓	✗	✗	✗	✗	✗	✗	✓	✓	✓	✗	✓	✓	✗	✗	✗	✗
Gas systems	✓	✓	✓	✗	✗	✗	✓	✗	✗	✗	✗	✗	✓	✓	✗	✗	✗	✗	✗	✗	✗	✗	✓	✗	✗
Rainwater drainage	✗	✗	✗	✓	✓	✗	✗	✗	✗	✗	✗	✗	✗	✗	✗	✗	✗	✗	✗	✗	✗	✗	✗	✓	✗
**E. Kampung Luar Batang**																									
Forms of water supply	✓	✓	✓	✗	✓	✗	✗	✓	✓	✗	✓	✓	✓	✗	✓										
Waste water sewage	✓	✓	✓	✗	✓	✗	✗	✗	✓	✗	✓	✗	✗	✗	✗										
Problems with waste collection	✗	✗	✓	✓	✓	✗	✓	✗		✗	✗	✓	✗	✗	✗										
Forms of electricity connection	✗	✗	✓	✓	✓	✓	✗	✗	✓	✗	✗	✗	✓	✗	✗										
Cable, internet, telephone, TV	✗	✗	✓	✓	✗	✗	✓	✗	✗	✗	✗	✗	✓	✗	✓										
Gas systems	✗	✗	✓	✓	✗	✓	✗	✓	✗	✗	✗	✓	✗	✗	✗										
Rainwater drainage	✗	✗	✗	✗	✓	✓	✓	✗	✗	✓	✗	✗	✗	✓	✗										
**F. Kampung Muara Baru**																									
Forms of water supply	✓	✗	✗	✓	✓	✗	✗	✗	✓	✗	✗	✗	✗	✓	✗	✓	✓	✗	✗	✗					
Waste water sewage	✓	✓	✓	✗	✗	✓	✓	✗	✓	✓	✓	✓	✗	✗	✗	✓	✗	✗	✓	✓					
Problems with waste collection	✓	✓	✓	✗	✗	✓	✓	✗	✗	✓	✗	✓	✗	✓	✓	✓	✗	✓	✗	✓					
Forms of electricity connection	✗	✓	✓	✗	✓	✗	✓	✗	✗	✗	✓	✓	✗	✓	✓	✗	✗	✗	✓	✗					
Cable, internet, telephone, TV	✗	✓	✓	✗	✗	✗	✗	✓	✗	✗	✓	✗	✓	✗	✓	✗	✗	✗	✗	✗					
Gas systems	✗	✗	✗	✓	✗	✗	✓	✗	✗	✗	✓	✓	✗	✗	✗	✗	✗	✗	✗	✗					
Rainwater drainage	✗	✗	✓	✗	✗	✗	✗	✗	✗	✗	✓	✓	✗	✗	✗	✓	✗	✗	✗	✗					

✓: Responder agrees with this feature.

✗: Responder disagrees with this feature.
