# Supplementary material for: The urban features of informal settlements in Jakarta, Indonesia
Source: Data Brief. 2017 Oct 24;15:993–9. doi: 10.1016/j.dib.2017.10.049 (PMC5684097; doi:10.1016/j.dib.2017.10.049)
Supplement: Supplementary file 1 — Supplementary material [file mmc1.docx]

**There is no conflict of interest**
